# Utilization of Kotter’s Stages and Statistical Process Control to Implement and Sustain Delirium Screening in PICU

**DOI:** 10.1097/pq9.0000000000000536

**Published:** 2021-12-07

**Authors:** Megan Kupferschmid, Sandeep Tripathi

**Affiliations:** From the Pediatric Intensive Care Unit, OSF Healthcare Children’s Hospital of Illinois, Peoria, Ill.

## Abstract

Delirium is a preventable cause of ICU morbidity and mortality. Prior unstructured efforts to implement delirium screening in our hospital were unsuccessful. This project aimed to improve the delirium screening compliance from baseline 0% to more than 80% within 12 months (07/2019–06/2020).

**Methods:**

We utilized Kotter’s stages of transformation as guiding blocks for implementing change and undertook simultaneous efforts to decrease delirium rates. In addition, we used statistical process control to monitor screening compliance and delirium rates.

**Results:**

Education on delirium assessment and prevention created a sense of urgency among nursing and medical staff (Stage #1). Stakeholder analysis and a key driver diagram were presented to leadership to create a guiding coalition (Stage #2). Process mapping and failure mode and effect analysis created a vision and strategy for the project (Stage #3). Multiple methods were utilized to communicate the change vision (Stage #4) and empower broad-based action (Stage #5). We celebrated small incremental achievements or short-term wins (Stage #6) by reporting delirium compliance and rate on a control chart. We completed 5837 delirium screens on 763 patients (6689 opportunities). The median delirium screening compliance rate was 87.2% (13.4% positive screens). Small achievements produced by the project were communicated to staff, propelling future efforts to recognize and prevent delirium. This momentum was a driver for more change (Stage #7) to ensure a long-term cultural change in the unit (Stage #8).

**Conclusion:**

Kotter’s principle of change management, along with an emphasis on data-driven process control, may result in sustained improvement.

## Abstract 2

Supplemental Digital Content is available in the text.

## INTRODUCTION

Pediatric delirium is a preventable and avoidable cause of ICU morbidity and mortality,^1,2^ with its prevalence in the PICU estimated to be up to 17%.^2^ Due to the lack of a standardized assessment method, pediatric delirium was unrecognized and underappreciated.^3,4^ Only recently have a few quality improvement (QI) projects focusing on delirium screening and early mobility been published.^5–7^ In 2015, OSF HealthCare Children’s Hospital of Illinois (CHOI) leadership recognized the need to assess delirium in children, and we implemented a delirium screening protocol using the Pediatric Confusion Assessment Method – ICU (pCAM-ICU).^8^ However, the ICU staff did not accept and utilize this process. While we did not access the exact etiology for the lack of sustained screening implementation acceptance at that time, prior investigators have cited the lack of change management,^9^ and utilization of methods focusing on education and process change alone,^10^ as critical causes of the failure of QI initiatives. These factors may also have contributed to the failure of our prior efforts to implement a delirium screening tool.

An organization can achieve a sustainable change when employees recognize a practice change provides significant value to the patient and themselves. Quality scholars have recognized this difficulty and have described various change management models.^11^ A widely utilized change management model is Kotter’s eight stages of transformation.^12,13^ Since its first description in the 1990s,^13^ Kotter’s change management principles have been successfully applied in healthcare and other service industries.^14–16^

Within this context and to avoid this common pitfall with QI initiatives, PICU leadership approached delirium screening and prevention with a systematic data-driven process by including Kotter’s change management principles. This article describes our processes of adopting Kotter’s principles into a PICU microsystem to affect employee behavior on delirium screening on critically ill children. The SMART aim of this project was to implement a new screening tool (Cornell Assessment of Pediatric Delirium [CAPD])^17^ in the pediatric ICU starting from 07/01/2019 and to achieve an 80% screening compliance rate with delirium screening on all eligible patients by 10/31/2019, and to sustain this rate through 06/30/2020.

## METHODS

### Context and Ethical Approval

We conducted a prospective mixed-methods study to implement delirium screening in the PICU. We created a multidisciplinary team, including a physician (ST) and a nurse educator (MK) as process owners, to achieve this change. This team was composed of nurses, physicians, APNs, and pharmacists. In addition, we wrote a formal project charter, which was reviewed and approved by the University of Illinois College Of Medicine Institutional Review Board as a quality improvement (QI) project.

### Setting

The Pediatric Intensive Care Unit (PICU) at the Children’s Hospital of Illinois (CHOI) at Peoria is a 16-bed multidisciplinary ICU that admits ≈1000 patients /y, including postoperative cardiac and level 1 trauma patients. PICU at CHOI is staffed 24×7 by board-certified/eligible pediatric intensivists, Advanced Practice Nurses, and Pediatric Residents.

### Interventions

We designed this process within the basic framework of the eighth stage change process as described by Kotter (**Supplemental Digital Content [SDC] Combined, figure #1** http://links.lww.com/PQ9/A360).

*Stage One: Establishing a Sense of Urgency*. Because of the lack of prior delirium screening, we lacked actual delirium incidence for our ICU. Thus, the staff perceived no visible crisis. Therefore, we used external evidence to generate urgency and drive staff out of their comfort zones. We surveyed PICU staff and families to establish a baseline of satisfaction with the current efforts to prevent and treat delirium in the PICU (SDC Combined, tables #2 and #3 http://links.lww.com/PQ9/A360). We further conducted a delirium knowledge assessment test to assess the current understanding of screening and treating delirium among the nursing and medical staff (SDC Combined, table #4 http://links.lww.com/PQ9/A360).

*Stage Two: Creating the Guiding Coalition.* Although initiating change is relatively easy, sustaining it requires the support of a powerful coalition. At the project’s onset, we conducted a stakeholder analysis^18^ to understand the needs and expectations of significant interests inside and outside the project environment (SDC Combined, table #5 http://links.lww.com/PQ9/A360). As a result, we included people of influence/position and power (nursing and medical director), people of high credibility among medical and nursing staff (MK as a nursing educator), and people with expertise and leadership to drive the change process (ST) in the project team. Together, this coalition had enough power to overcome the staff inertia. In addition, we selected the goal to be sensible (high-quality evidence exists that delirium is common in PICU patients^1,2^) and appealing (treating/preventing delirium can make patients more comfortable, require less sedation, and have better outcomes, all of which appeal to the PICU staff^19^).

*Stage Three: Developing a Vision and Strategy.* Kotter described vision as “a sensible and appealing picture of the future.”^12^ Our overarching vision during this process was to decrease the need for pharmacological sedation and get kids moving as soon as medically possible. We adapted this vision from the ongoing national efforts on improving PICU outcomes like ICU liberation^20^ and PICU Up^7^ projects. This vision statement also aligned with the six characteristics described by Kotter (imaginable, desirable, feasible, focused, flexible, and communicable).

**Fig. 1.**
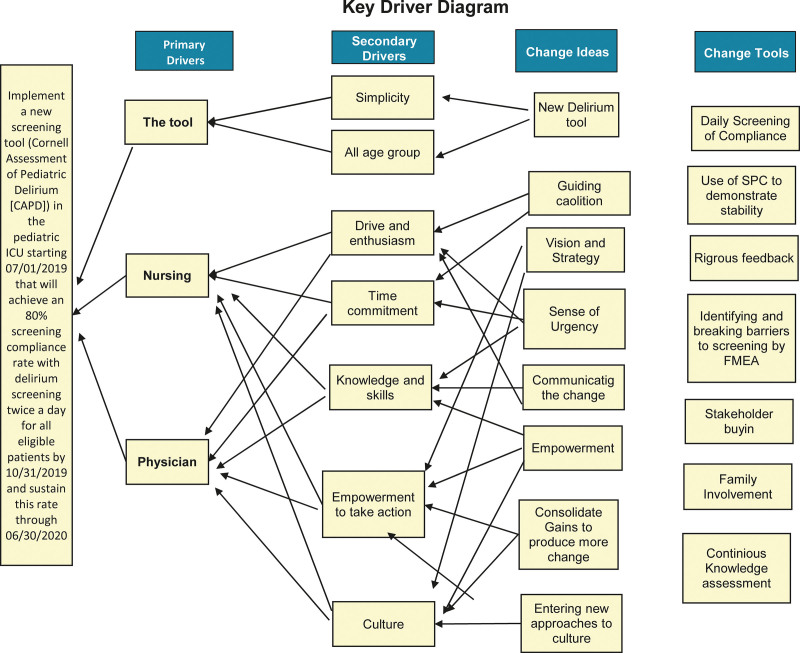
Key driver diagram.

**Fig. 2.**
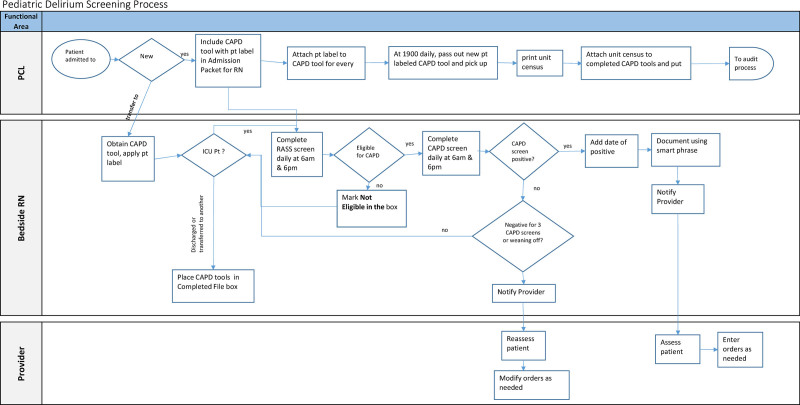
Process map for delirium screening and auditing. PCL, Patient Care Liaison a.k.a. unit secretary; ; ICU, Intensive Care Unit; RASS, Richmond Agitation Sedation Scale; RN, Registered Nurse.

**Fig. 3.**
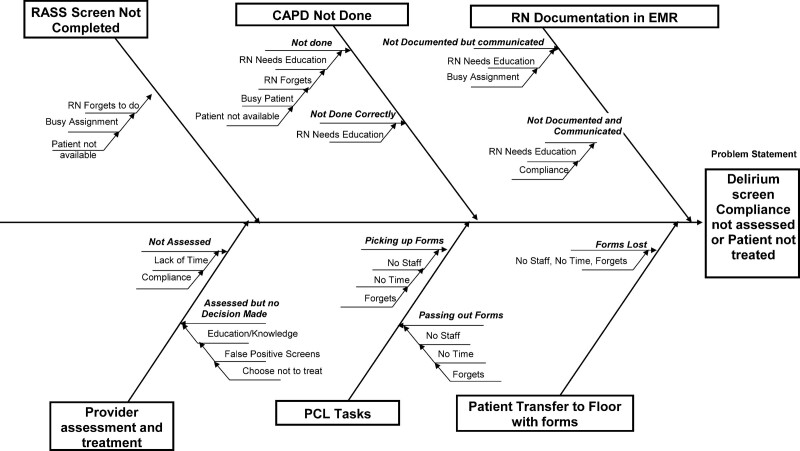
Fishbone diagram on potential causes of failure of delirium screen audit and patient evaluation. RASS, Richmond Agitation Sedation Scale; RN, Registered Nurse; ; PCL, Patient Care Liaison aka unit secretary.

Kotter defines strategy as “a logic and details on how goals can be accomplished.”^12^ Our strategy for this project focused on operational methods and monitoring of the processes to remove nonconformity causes at all stages of the delirium screening process. We began the project implementation by creating a Key Driver Diagram (Fig. 1) and Process map (Fig. 2). The process map delineated each staff member’s necessary functions to screen a PICU patient for delirium successfully. We utilized this process map to create a Fishbone diagram on potential areas of failure (Fig. 3). We then conducted a Failure Mode and Effect Analysis (FMEA) to identify areas where the system might fail and delirium screening would not occur (**SDC Combined, table #6**. http://links.lww.com/PQ9/A360).

Based on the FMEA, we estimated the potential effects of failure, the severity and possible causes of the failures, the estimated rate of occurrence, and the ability of current controls to prevent failure by consensus. We then calculated the risk prediction number for all process steps. The highest risk prediction number number (315) was for “provider assessment and treatment decisions within 2 hours of detection of delirium by nursing.” The second highest risk prediction number number (252) was “loss of delirium screening forms when the patient was transferred from pediatric ICU to the pediatric floor.” We prioritized these high RPM categories during the project’s design and implementation (**SDC Combined, table #6.** http://links.lww.com/PQ9/A360).

*Stage Four: Communicating the Change Vision.* As per Kotter’s principles, we wanted success to be visible, well communicated, and proven.^12,21^ We developed multiple strategies to show and celebrate short-term wins. For our project, we sent monthly emails to staff to inform them of their monthly screening compliance. In addition, MK (ground-level change agent for the process) did rounds on the unit at delirium screening time to remind staff to complete their screens. The communication strategy utilized followed the essential elements described by Kotter,^12^ including (1) simple, (2) use of analogy and metaphor [case-based education], (3) multiple forum/sources, (4) repeated and consistent communication, (5) leadership by example (project team members scored the highest compliance), and (6) explanation of seeming inconsistencies (eg, moving kids out of bed, but decrease unplanned extubation rates).

**Fig. 4.**
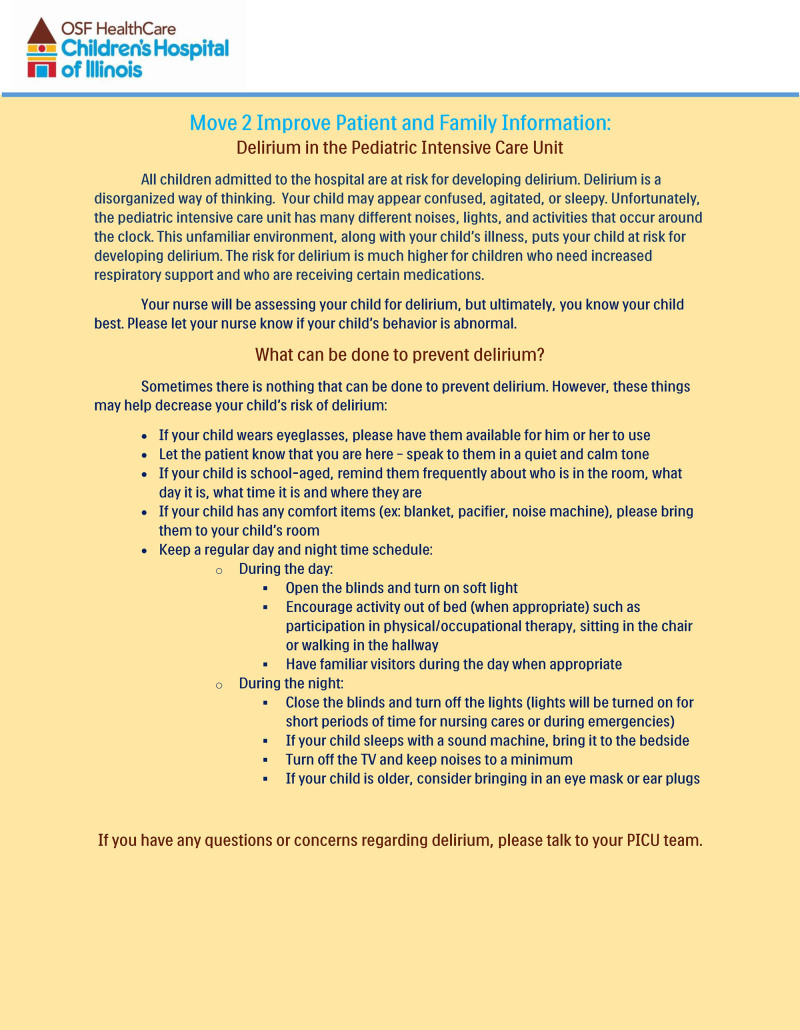
Family information brochure.

*Stage Five: Empowering Broad-Based Action.* Kotter described empowerment as “helping people become more powerful.”^12^ In this project, we accomplished this by giving people the skills to measure and prevent delirium. We also removed structural barriers in assessing delirium by providing screening charts at the bedside and later by implementing the CAPD scores in the EMR. We encouraged the nurses to write a note in the chart with the positive screen with the physician’s name and action taken. As part of empowerment, we created education for nursing and medical staff. We educated medical staff on using the CAPD and necessary measures when notified of a positive screening result. Additionally, we included families as care partners in the project and created informational literature regarding pediatric delirium (Fig. 4).

*Stage Six: Generating Short-Term Wins.* According to Kotter, people need to see convincing evidence that their efforts are paying off. Therefore, we planned to document the visual improvement in the process or “wins.” Although our overall vision for this project was to decrease delirium and improve patient outcomes, our short-term focus was monthly screening compliance. Monthly compliance goals created more wins for staff, thus driving the change. We also provided recognition to the nurses who achieved high compliance scores.

*Stage Seven: Consolidating Gains and Producing More Change.* Successful implementation of any change in healthcare depends on the involvement of all healthcare team members. With this project, a nurse would identify patients with a positive delirium screen and contact the physician. The physician would assess the patient and then take action based on their assessment. A breakdown in this multi-step process would lead to decreased compliance. To ensure this step, we introduced training of physicians and established protocols to produce an ongoing change. People involved in the process were recognized with awards (‘*Nurse Innovator*’ award to MK), and funding was secured to purchase two in-bed cycle ergometers (RT300 supine, Restorative Therapies Inc, Baltimore MD) to continue our efforts to increase mobility and decrease delirium.

*Stage Eight: Anchoring New Approaches in the Culture.* To complete Kotter’s steps for change management, we understood that delirium assessment would need to be embedded into the unit’s “norms of behavior” and “shared values.” If the unit’s culture is not changed, staff will revert to old habits as soon as the transformational agent gets sidetracked or is replaced. We believe that our unit’s culture has changed because we were able to alter people’s actions (assess delirium regularly), which led to group benefit (high compliance and decrease in delirium rates), for a sustained period, which made staff realize the connection between their new actions and improvement.

### Process and Outcome Measures

*Delirium Screen compliance.* The project focused on the monitoring of delirium screens on all eligible patients. Before the inclusion of delirium screening scores in the EMR (07/01/2019 to 11/09/2019), the bedside nurse completed the delirium screening on paper twice a day (SDC Combined, figure #7 http://links.lww.com/PQ9/A360). We then manually compiled the screens daily and compared the number of screens to the unit census to calculate the screening compliance rate. After including delirium screening scores in the EMR, the nurse completed the delirium screening in the EMR; however, we still did the compliance monitoring manually. The CAPD is expected to be performed twice daily on all patients in the PICU. In this project, the screening compliance was measured based on the morning census in the PICU. Screening opportunities were calculated as 1.6× the morning census to account for the discharges during the day and allowance for missing paper screening (rounded to the nearest integer). We tolerated some inaccuracy in this rate as we expected this to balance out over the year. It was also possible for the compliance rate to be > 100% on certain days. We created a p-chart of the weekly compliance with 3σ upper and lower control limits (UCL & LCL) based on weekly audits to monitor process stability. To ensure personal accountability for delirium screening, we measured the delirium opportunities and completed screens for every nurse in the pediatric ICU every month. This approach also created healthy competition among nursing to achieve the highest compliance rate.

*Delirium Rates.* We compiled the incidence of positive delirium screens in the PICU and reported them as a p-chart of the fraction of positive screens out of total screens for the week.

*Staff self-assessment of the measures to prevent delirium in their patients.* We conducted a staff self-knowledge assessment on 20 random nurses every month for the project’s duration (**SDC Combined, table #2.** http://links.lww.com/PQ9/A360).

*Objective Knowledge Score.* We conducted a 10-question knowledge assessment test before and after staff education and after 12 months to assess knowledge retention. (**SDC Combined, table #4.** http://links.lww.com/PQ9/A360.)

*Family Satisfaction Surveys.* To assess the family satisfaction with the delirium screening and prevention measures, we surveyed the parents and caregivers of patients admitted to the ICU before and after implementation. (**SDC Combined, table #3.** http://links.lww.com/PQ9/A360.)

## STATISTICAL ANALYSIS

We performed standard descriptive analysis, including calculating the median and interquartile range for continuous variables and frequency (percentage) of categorical variables. In addition, we conducted the comparative analysis using Chi-square/Fischer exact test or Kruskal Wallis test as appropriate and calculated the correlation coefficient between delirium screening compliance and rates of the positive screen. We used JMP V.14.8 (SAS Institute, Cary, N.C.) for all statistical analysis and QI. Macros (KnowWare International, Inc, Denver, CO) for designing QI charts and graphics.

## RESULTS

A total of 763 patients were admitted to the PICU during the project period. Of these patients, we audited a total of 5837 delirium screens, out of 6689 possible opportunities (1.6×patient days), with a cumulative median delirium screening compliance rate of 87.2%. Overall delirium screen positive rate was 13.4% (783/5837).

### Delirium Screen Audits

**Fig. 5.**
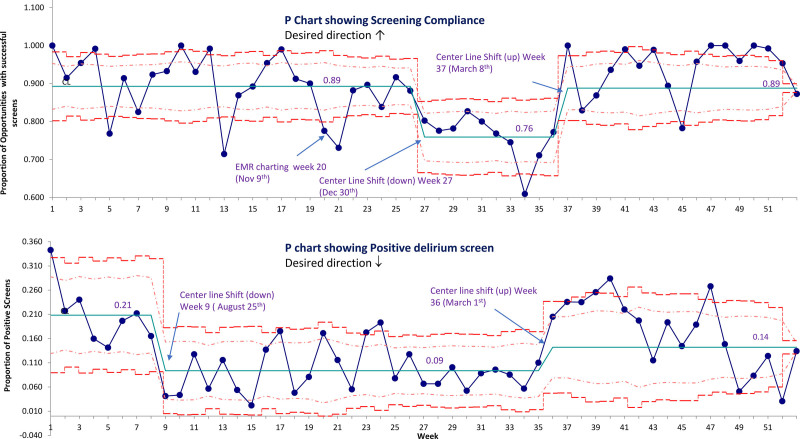
p-Chart of weekly delirium screening compliance and positive delirium screens. Upper and lower limits represent 3σ. Screening compliance represents the number of screens completed out of total opportunities (1.6× morning census of the PICU. Compliance capped at 100%). The positive delirium screen represents the fraction of positive screens out of total screens for the week.

The P chart of the screening compliance had a centerline of 0.89. There was a downward centerline shift from week 27 to week 36 (December 29, 2019, to March 7, 2020) with a return to baseline from week 37 onwards. The process had a few weeks with compliance below the LCL (week 5, 13, 20 – 21, 34, and 45) and 12 weeks with compliance higher than the UCL. The overall lowest compliance was observed on week 34 (February 16–22, 2020). Some of the out-of-control weeks could be explained by special cause events (introduction of EMR charting on week 20, and high PICU census with the increased nursing workload during the busy winter months leading to low compliance; while increased awareness during the World Delirium day celebrations on March 11, 2020 [week 37]^22^ inspiring high compliance). No specific cause could be identified for other out-of-control events (Fig. 5). The monthly delirium screening compliance remained more than the goal (80% throughout the project, except for February 2020), with the lowest median screen compliance of 71% (IQR 67%, 77%). Screening compliance steadily increased after that, with a 104% (IQR 95%, 112%) compliance in June 2020 (SDC combined, figure #8 http://links.lww.com/PQ9/A360). Two nurses (3.1%) obtained 100% compliance with delirium screening throughout the twelve months of the project, and 15 nurses (23.4%) had more than 90% compliance. (SDC combined, figure #9 http://links.lww.com/PQ9/A360).

### Positive Delirium Screens

The P chart for the proportion of positive delirium screen showed the initial centerline of 0.21 with a downshift to 0.09 by week 9 (August 25, 2019). The rate of positive delirium screens remained stable up to week 36, after which there was a centerline shift upwards to 0.14. A few weeks had rates of positive delirium screens beyond the upper control limit (notably the 1^st^ week of the project, week 24, and week 40 and 46). The upward shift of the centerline of positive screens from week 36 (along with a run of 8 points above the centerline from week 35–42) coincided with the increased delirium screening compliance from week 37 onwards; however, no specific cause of the other out-of-control weeks could be identified (Fig. 5). The monthly percentage of positive delirium screens was highest at 22% (IQR 13.6%, 30%) in the first month of implementation, while it was lowest in the last month of the project (06/20, 5%, IQR 0%, 9.3%) (SDC Combined, figure # 10 http://links.lww.com/PQ9/A360). There was an overall low positive correlation of positive delirium screen with screening compliance (Correlation coefficient 0.18 [95% CI 0.07, 0.27]).

### Staff Self-Assessment Surveys

**Fig. 6.**
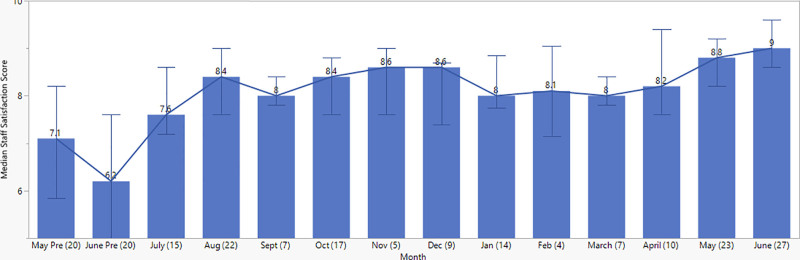
Median cumulative staff self-assessment score by month, out of maximum 10. Data include 2 months of pre-implementation assessment and 12 months of change management. Values represent the median and interquartile range (IQR). *x* axis values in parentheses represent the sample size of the respective month. Median pre-implementation score 7.0 (IQR 5.4, 8.0)/10 and post-implementation score 8.4 (IQR 7.8, 9.0)/10, *P* < 0.001

The monthly median self-assessment score for the staff remained consistently above 8 throughout the implementation phase after the first month (Fig. 6). The cumulative score showed a significant improvement from the pre-implementation phase (7.0 [IQR 5.4, 8.0]/10 [n = 40]) to the post-implementation phase (8.4 [IQR 7.8, 9.0]/10 [n = 160]), *P* < 0.001 (Fig. 6). Among the five domains of self-assessment, the staff gave themselves the lowest score in the “knowledge to screen” and “knowledge to treat.” These numbers showed steady improvement throughout the project. (**SDC Combined, table #11.** http://links.lww.com/PQ9/A360).

### Objective Knowledge Score

The mean cumulative test score improved from 5 (IQR 4, 7)/10 in the pre-test to 9 (IQR 8, 9)/10 in the post-test (*P* < 0.01). We observed a significant increase in the cumulative score one year after the project implementation from the pre-test (*P* < 0.01) but not from the post-test administered immediately after the staff education (*P* = 0.64). We observed maximum improvement in the knowledge in questions that pertained directly to the project, including “which screening results indicate a positive delirium score on the CAPD” (17.2%, 86.9%, and 81.8%, *P* < 0.01) and “what RASS scores render the patient ineligible for delirium screening” (40.0%, 95.6% and 94.4%, *P* < 0.01) (SDC Combined, table #12 http://links.lww.com/PQ9/A360).

### Family Satisfaction Survey

Cumulative family satisfaction scores increased from a median of 7.7/10 (IQR 5.3, 8.5) (n = 11) to 8.8/10 (IQR 8.2, 9.6), (n = 10), *P* = 0.01. Families showed much higher satisfaction with every delirium prevention and treatment element in the post-implementation phase, with the maximum difference observed by the family in the involvement of child life (6.4/10 versus 9.6/10) and bath/linen change in the daytime (6.2/10 versus 8.0/10) (**SDC Combined, figure #13.** http://links.lww.com/PQ9/A360).

## DISCUSSION

This report described the implementation of a delirium screening tool in the PICU with a robust data-driven process. To the best of our knowledge, this is the first description of the use of Kotter’s principles of change management for the process of delirium screening in the pediatric ICU. The defining feature of this endeavor provides context, challenges, and solutions to implementing change in the PICU setting.

While there are multiple models of change management,^23^ we utilized Kotter’s principles in this project as we find them intuitive and scholarly adaptive.^11,24^ A recent systematic review identified 38 published QI projects on change management, 19 (50%) utilized Kotter’s principles. Kotter’s principles can be adapted to different environments and are suitable for microsystem changes by frontline leaders like our project.^11^ As seen with prior reports,^24^ this project utilized Kotter’s principles as a guide map rather than a strict recipe. Kotter’s model provides limited guidance concerning influencing people with resistance to change or changing people’s behavior.^25^ Recognizing this limitation, we supplemented this project with SPC and other visual data presentation tools (ie, process map, FMEA) to encourage change. Kotter also suggests that change agents follow all of his eight steps in sequence. Although the team attempted to avoid overlapping, it was not always feasible. Other scholars have previously discussed this rigid approach of Kotter as a limitation.^15^

Statistical process control (SPC) throughout the process is essential for any QI project. The results of this project align with Simone et al, who showed a sustained improvement after implementation of an ICU bundle, including delirium, using SPC (decrease in delirium rates from 19.3% to 11.8%).^6^ We utilized both delirium screening and the rate of delirium as a control chart for the project’s duration. Although there were many weeks where the screening compliance and rate of positive delirium were out of control, we could identify such events and initiate remedial efforts to stabilize the process because of the use of SPC. We observed the lowest screening compliance in the busy winter months and overall lower rates of positive delirium screens during those months. Higher screen compliance in subsequent months was accompanied by increased identification of delirium. Even though low, the correlation of screening compliance with the rates of positive delirium screens was positive. This finding probably reflects the subtle nature of delirium in pediatrics. Due to the time constraints of patient care, nurses are more likely to miss a delirium screening in a patient with high acuity,^5^ thus driving the overall delirium prevalence rate down.

As is often reported in QI projects, our biggest challenge in this project was change fatigue.^26^ In addition, multiple QI projects are often being implemented simultaneously in a busy ICU, sometimes with competing interests and goals. We attempted to overcome this by creating a shared vision for the unit, which aligned all the QI projects. Having a shared vision also impacts change fatigue, as staff are more likely to accept change if the strategic direction is similar.^27^

Our project had several limitations. First, our delirium screening process and delirium rates were not stable throughout the project, with many out-of-control weeks. Second, even with a sustained screening implementation, we did not significantly decrease the delirium rate throughout the project. However, our emphasis was on the importance of delirium screening and recognition by the staff, including physicians. Third, the questionnaire, tests, and surveys used in this project are not validated. Fourth, like other change management projects, our project does not differentiate the efficacy of one improvement model over the other. Even though we were previously unsuccessful in implementing a delirium screening tool that produced lasting change, it is unclear whether the tool or the implementation strategy led to the project’s success this time. Fifth, in this project, we did not conduct any objective measurement for the resistance to change or reasons thereof. We also did not perform any quantification of the culture change. Sixth, our overall compliance rate may be overestimated due to appraisal compromises. And, lastly, very few family satisfaction surveys were obtained due to the low response rate, limiting their interpretation.

## CONCLUDING SUMMARY

In conclusion, we have described a data-driven process change with the utilization of Kotter’s principles. Even though this is a single-center project, the challenges we face are not unique to us, and by writing this report, we hope to create awareness of the change model and implementation science. Further research on change management should focus on generating evidence of the effectiveness of different change management models and identifying which model may work best in different environments.

## DISCLOSURE

The authors have no financial interest to declare in relation to the content of this article.

## ACKNOWLEDGMENTS

We would like to thank Ms. Christine Hancock, CSSBB, for assistance in the design of FMEA, Ms. LaMonica Henrekin, RN, BSN, NE-BC (nurse manager, pediatric intensive care unit) for guidance and support of the QI team; Mr. Jeremy McGarvey, MS (Senior Statistician, OSF Healthcare, Peoria) in assistance in designing the control chart of screening compliance; and Dr. Mary R. Cooper (Program Director, Master’s Program in Health care Quality and Safety, Jefferson College of Population Health) in guidance toward Capstone project. We acknowledge all the staff members, including nursing, PICU secretarial staff, and medical staff, for their tireless efforts in improving the quality of care in the ICU.

## Supplementary Material


