# Supplementary material for: Utilization of Kotter’s Stages and Statistical Process Control to Implement and Sustain Delirium Screening in PICU
Source: Pediatr Qual Saf. 2021 Dec 7;6(6):e536. doi: 10.1097/pq9.0000000000000536 (PMC8654424; doi:10.1097/pq9.0000000000000536)
Supplement: Supplementary file 1 [file pqs-6-e536-s001.pdf]

Utilization of Kotter's stages of transformation and statistical process control to implement and sustain delirium screening in the PICU

Supplementary Digital Content

Megan Kupferschmid, MSN, RN, Sandeep Tripathi, MD, MS

Table of Contents

No	Table /Figure	Page No
1	Project Infographic/Visual abstract	2
2	Staff self-assessment survey questions	3
3	Family satisfaction survey questions	4
4	Staff pre-and Post-test questions	5
5	Stakeholder analysis	6
6	FMEA	7
7	Paper delirium screening form	9
8	Monthly delirium screening compliance	10
9	Percentage compliance by nursing	11
10	Monthly positive delirium screens	12
11	Staff self-assessment survey results	13
12	Staff pre-and post-test results	14
13	Family Satisfaction survey results	15

SDC #1 Infographic/Visual Abstract

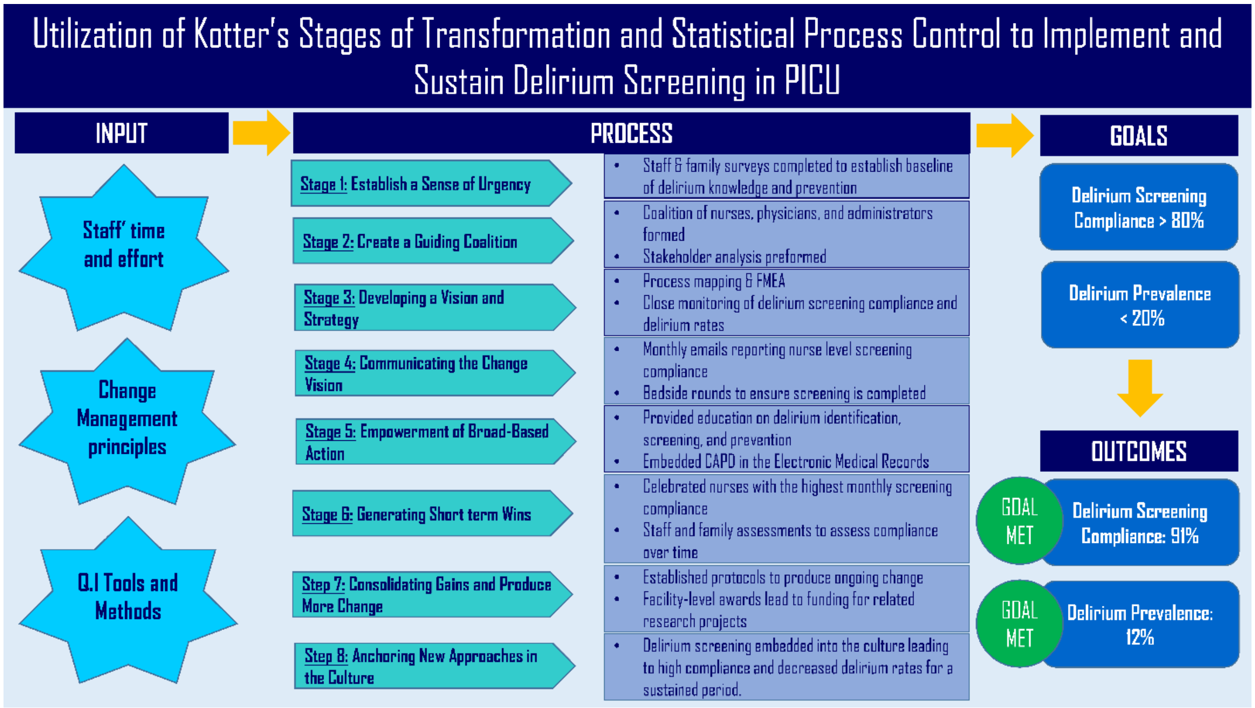

SDC #2: Staff self-assessment survey questions

	Please rank on a scale of 1 to 10 were one is least and 10 is highest/optimal										
1	How comfortable do you feel regarding your knowledge to screen for delirium on your patient?	1	2	3	4	5	6	7	8	9	10
2	Did you modify the patient environment to support a normal sleep-wake cycle?	1	2	3	4	5	6	7	8	9	10
3	If you're patient were to screen positive for delirium who during your understanding of the actions, you will take to treat delirium	1	2	3	4	5	6	7	8	9	10
4	How would you rank your understanding of the nonpharmacological interventions that can reduce delirium in a patient	1	2	3	4	5	6	7	8	9	10
5	How would you rank the noise level for your patient	1	2	3	4	5	6	7	8	9	10
6	How would you rank child life specialist involvement in your patient care	1	2	3	4	5	6	7	8	9	10
7	How would you rank the physical rehabilitation team's involvement in your patient care	1	2	3	4	5	6	7	8	9	10

SDC #3: Family satisfaction survey questions

	Rank on a scale of 1 to 10 where one is less, and 10 is maximum (best)										
1	When your child was in the ICU were the lights in the room dimmed at night as much as possible	1	2	3	4	5	6	7	8	9	10
2	When your child was in the ICU how was the noise level value child was sleeping	1	2	3	4	5	6	7	8	9	10
3	When your child was in the ICU, was a child life specialist involved in your child's plan of care	1	2	3	4	5	6	7	8	9	10
4	When your child was in the ICU, was to pediatric rehabilitation team involved in your child's plan of care	1	2	3	4	5	6	7	8	9	10
5	When your child was in the ICU, was their staff members assisting your child to sit in a supported seated position three times a day or more	1	2	3	4	5	6	7	8	9	10
6	When your child was in the ICU, did staff attempt to use soothing techniques other than medications to help ease pain/anxiety/fear for your child	1	2	3	4	5	6	7	8	9	10
7	When your child was in the ICU, did the staff do a bed and Linen change between the hours of 5 AM and 11 PM (Day time)	1	2	3	4	5	6	7	8	9	10
8	If your child wears glasses, did staff ask you to bring them to your child's bedside										
9	If your child has a comfort item (pacifier/blanket/book et cetera) did staff ask you to bring them to your child's bedside										

SDC #4: Pre and post test questions

Question	Choice 1	Choice 2	Choice 3	Choice 4
Approximately what percentage of pediatric ICU patients are thought to experience delirium	5%	10%	25%	50%
The age group with the highest incidence of pediatric delirium is (Years)	0-2	3-6	7-11	12-18
CAPD scale is applicable to patient's age	> 5	3-18	0-21	12-18
How often is the CAPD scale performed during a 24 hour period	Once/day	Twice/day	Q2 hours	Q1 hour
What score indicates a positive delirium screen with the CAPD scale	5	8	9	11
Which RASS would exclude patients from the CAPD	+3	0	-5	+5
Which of the following is not a risk factor for patients who develop pediatric delirium	Developmental delay	Age	Gender	Illness severity
Which classification of medication is most likely to cross pediatric delirium	Antibiotics	Benzodiazepines	Inotropes	Opioids
Which of the following is not an intervention to prevent pediatric delirium	Limiting visitors	Turning on lights/shades by 9 AM	Avoid bed/linen change during sleeping hours	Provide comfort item from home
Which medication can be used to treat pediatric delirium	Midazolam	Risperidone	Methadone	Klonopin

SDC #5: Table Stake Holder Analysis

	Stakeholder		Interests	Project influence	Project importance	Assumptions
Primary	Owner	Physician lead (ST) and nursing educator (MK)	Achieve targets	+++	Critical	Time and conflicting projects
	Sponsor	Nursing and medical director	Alignment with hospital policies	+	Critical	Return of investment
	Team members	Nursing	Patient comfort and safety	++	Critical	New product excitement
		Medical staff	Diagnosis and management	+	Modest	Liability
		Physical therapist	Patient comfort mobility	+	Minimal	Retain and expand the skillset
Secondary	Secondary stakeholders	Patient/family	Patient comfort, safety, and improvement	+++	Critical	Patient comfort would be increased
		Secretarial staff	Unit workflow	+	Minimal	Continued engagement
		Information technology	Hospital workflow	+	Minimal	Time commitment
		Pharmacy	Medication uses	+	Minimal	Medication availability
		Neurology/psychiatry	Accurate diagnoses	+	Minimal	Timely consults
		Child life	Patient comfort	+	Modest	Time commitment and engagement

SDC #6: Failure Mode and Effect Analysis

Process steps	Potential Failure Mode	Potential Effects of failure	Severity	Potential causes of failure	Occurrence	Current Controls	Detection	RPN
RASS Screen Completed	Not Done	Patient	9	RN Forgets to do	3	PCL Picks up	3	81
			9		3	Weekly audit process	5	135
			9	Patient assignment too busy	3	Asks for Help	5	135
			9	Patient not available	1		1	9
CAPD	Not done	Delirium not identified	9	RN needs education	4	Weekly audit process	5	180
				RN forgets to do	4	PCL picks up	3	108
					4	Weekly audit process	5	180
				Busy patient	3	Ask for help	5	135
				Patient not available	1		1	9
	Not done correctly	Delirium not identified	5	RN needs education	2	Weekly audit process	7	70
RN documentation in EMR	Not documented but communicated	Legalities	5	Needs education	3	Weekly audit process	5	75
			5	Busy patient assignment	4	Asked for help	5	100
	Not documented and not complicated	Lack of treatment	9	Education	1	Weekly audit process	3	27
			9	Compliance	3	Weekly audit process	7	189
			9	Busy patient assignment	3	Ask for help	5	135
Provider assessment and treatment within two hours	Not assessed	Lack of treatment	9	Lack of time	3	Bedside RN	3	81
			9	Compliance	5	Bedside RN	5	225
			9		5	Weekly audit process	7	315
	Assessed but no decision made	Lack of treatment	5	Education/knowledge	5	Weekly audit	7	175
			5	False positive screen	3	Attending physician	3	45
			5	Choose not to treat	5	Bedside RN	3	75
PCL task								
Picking up	No data for audit	No compliance measurement	3	No staff, no time, forgets	4	Weekly audit	3	36
Passing out	No form available for RN	Lack of assessment/screen not completed	9	No staff, no time, forgets	4	RN notices	3	108

Process steps	Potential Failure Mode	Potential Effects of failure	Severity	Potential causes of failure	Occurrence	Current Controls	Detection	RPN
Transfer to floor to get forms	No form available for RN	Lack of assessment/screen	9	No staff, no time, forgets	4	RN notices	7	252

SDC #7: Paper delirium screening from

OSF HealthCare
Children's Hospital
of Illinois

0600

Patient Sticker: **Date:**

Cornell Assessment of Pediatric Delirium (CAPD)						
**RASS Score:** *If -4 or -5: patient is ineligible for the screen*						☐ Ineligible
	Never	Rarely	Sometimes	Often	Always	Score
1. Does the child make eye contact with the caregiver?						
2. Are the child's actions purposeful?						
3. Is the child aware of his/her surroundings?						
4. Does the child communicate needs and wants?						
	Never	Rarely	Sometimes	Often	Always	Score
5. Is the child restless?						
6. Is the child inconsolable?						
7. Is the child underactive – very little movement while awake?						
8. Does it take the child a long time to respond to interactions?						

If your patient has a score of ≥ 9, your patient has a *positive* delirium score.

1. Notify the PICU ROC/APN/Intensivist of positive delirium score.
2. Put an interdisciplinary note with the smart phrase ".peddelirium"

SDC #8: Monthly Screening Compliance

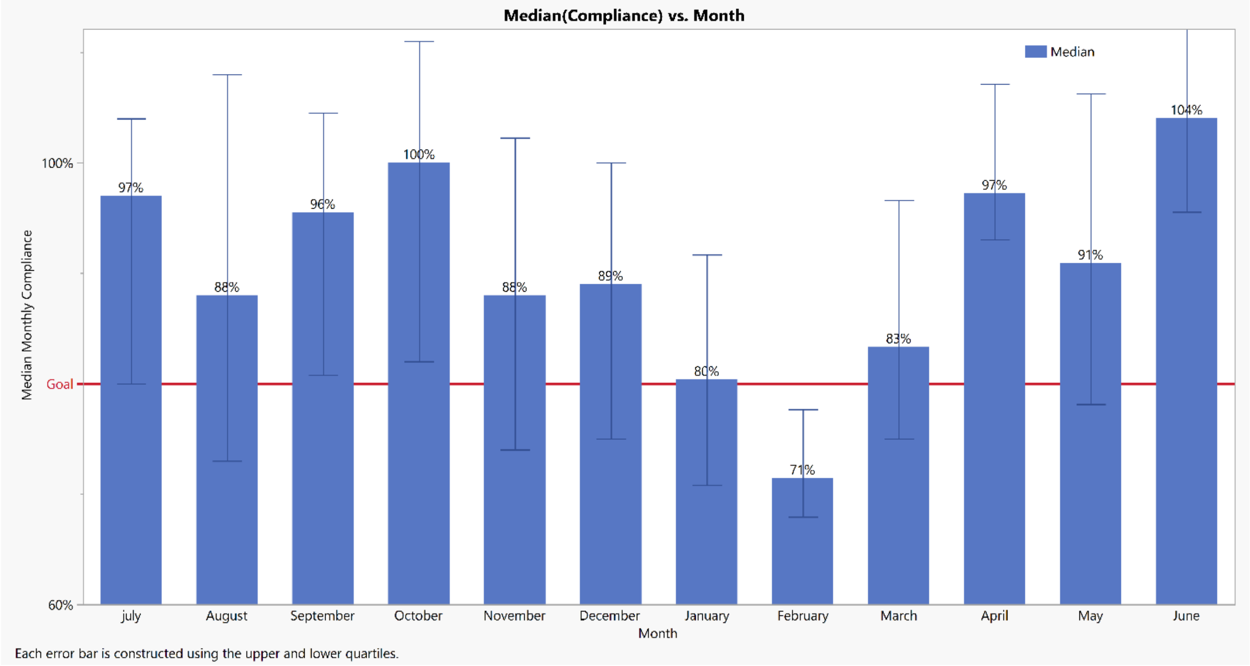

SDC #9: Histogram showing the number of nurses by the category of cumulative percentage compliance with delirium screening over one year

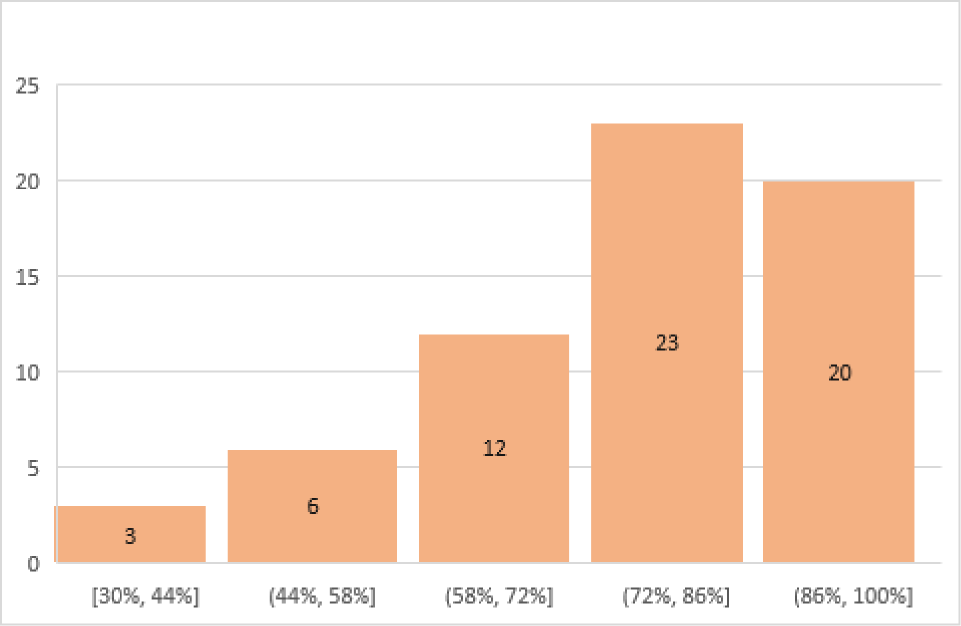

2 nurses (3.1%) had 100% compliance and 15 (23.4%) nurses had more than 90% compliance

SDC # 10: Monthly Positive Screens

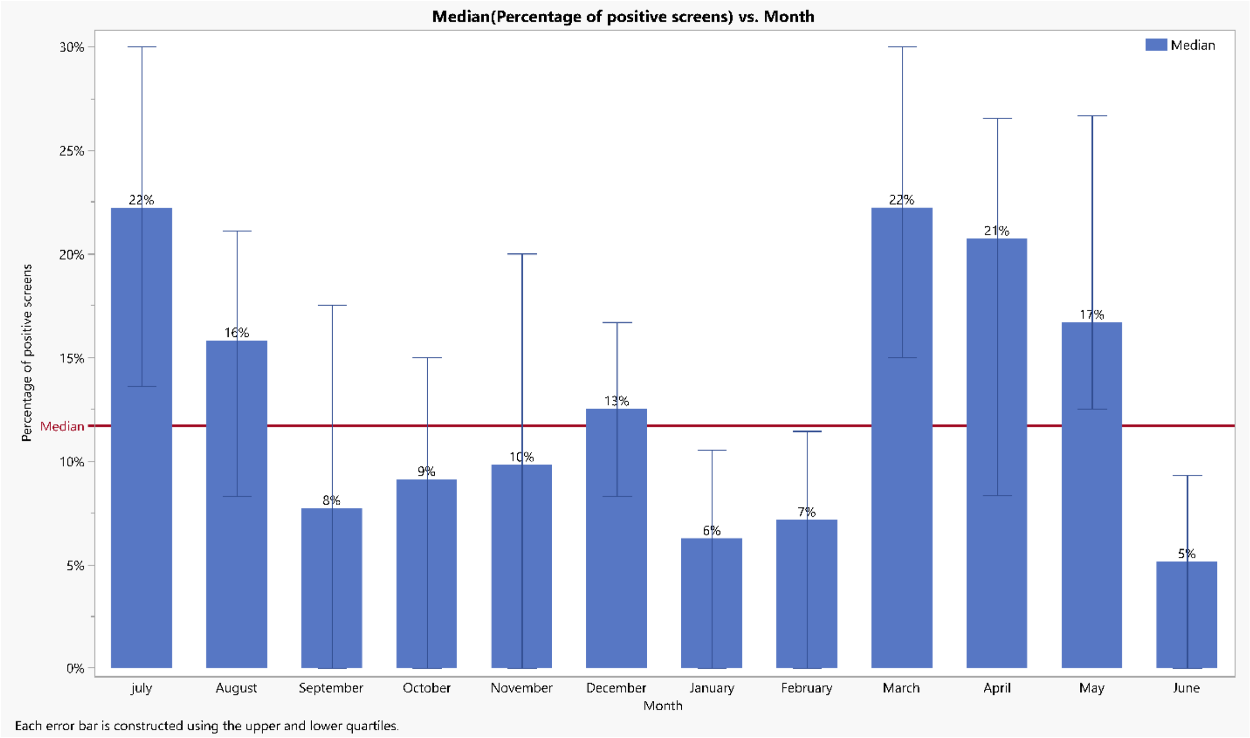

SDC # 11: Staff self-assessment on different domains of delirium prevention and treatment by month

	N	Knowledge to Screen	Modify Environment	Knowledge to Treat	Knowledge to Prevent	Noise Level
May Pre	20	6 (5, 8)	9 (7.2, 9.7)	6.5 (4.2, 8)	8 (6.2, 8.7)	7 (6, 8)
June Pre	20	5.5 (3, 8)	8 (6, 9)	6 (4, 8)	6.5 (3.2, 8)	7 (4.2, 8)
July	15	8 (7, 10)	9 (8, 10)	8 (5, 9)	8 (7.7, 9.2)	7 (6, 9)
August	22	9 (8, 10)	9 (7, 10)	8.5 (6.7, 10)	9 (8, 10)	7 (5.7, 9)
September	7	9 (8, 10)	9 (7, 10)	8 (6, 9	8 (7, 9)	8 (4, 9)
October	17	9 (7.5, 10)	9 (8, 10)	9 (9, 10)	9 (8, 10)	7 (5, 8.5)
November	5	10 (9, 10)	8 (7.5, 9.5)	9 (9, 10)	10 (7.5, 10)	7 (3, 7)
December	9	9 (8, 10)	8 (7.5, 10)	8 (7, 10)	8 (7.5, 10)	8 (5.5, 9)
January	14	9 (8, 10)	8.5 (7, 10)	8 (7.7, 10)	9 (8, 10)	7 (3.7, 8.2)
February	4	8.5 (6.5, 9.7)	8.5 (6.5, 9.7)	8.5 (5, 9.7)	9.5 (9, 10)	7 (3.7, 9.5)
March	7	9 (9, 10)	9 (7, 10)	9 (7, 10)	9 (7, 10)	7 (2.5, 9.2)
April	10	9 (8.5, 10)	9 (7.2, 10)	9 (8, 9)	9 (8, 9.2)	8.5 (5.7, 7)
May	23	10 (10, 10)	9 (8, 10)	10 (8, 10)	9 (9, 10)	8 (5, 8)
June	27	10 (10, 10)	10 (9, 10)	10 (8, 10)	10 (9, 10)	8 (7, 10)
P value		<0.001	0.28	<0.001	<0.001	0.20

Values represent median and inter quartile range. P value by nonparametric Wilcoxon/Kruskal Wallis test

SDC #12: Pre and post test results

		Pre-Test (n= 30)	Post Test (n= 23)	At 1 year (n= 22)	P-Value
Incidence of delirium in the ICU	% Correct	46.6%	69.5%	63.6%	0.20[c]
Age which has the highest delirium incidence	% Correct	26.6%	43.4%	27.2%	0.36[c]
CAPD applicable for which age group	% Correct	44.8%	100%	95.4%	<0.01[c]
How many times CAPD assessed in the day	% Correct	83.3%	100%	100%	0.01[c]
What CAPD score is positive for delirium	% Correct	17.2%	86.9%	81.8%	<0.01[c]
What a RAS score exclude CAPD assessment	% Correct	40.0%	95.6%	95.4%	<0.01[c]
Risk factor for development of delirium	% Correct	63.3%	82.6%	63.6%	0.25[c]
Medication most likely to cause delirium	% Correct	70.0%	95.6%	90.9%	0.02[c]
Interventions to prevent delirium	% Correct	63.3%	86.9%	90.9%	0.02[c]
Medications to treat delirium	% Correct	90.0%	100%	100%	0.09[c]
Cumulative correct score	Median (IQR)	5 (IQR 4, 7)	9 (IQR 8, 9)	8 (IQR 7, 9)	<0.001[k*]

**Nonparametric comparison using Dunn method for joint ranking showed significant difference between pre-and post-test (p<0.001) and between pre-test and score at one year (p<0.001). There was no significant difference between post-test and score at one year (p= 0.64)*

[c] *chi-square test*, [k] *Kruskal Wallis test*

[$] *62 (82%) nursing, 5 (6.6%) residents, 5 (6.6%) attending physician and 3 (4.0%) advanced practice providers*)

SDC #13: Before and after family surveys on structural and process changes being implemented in the unit to decrease the incidence of delirium in the ICU.

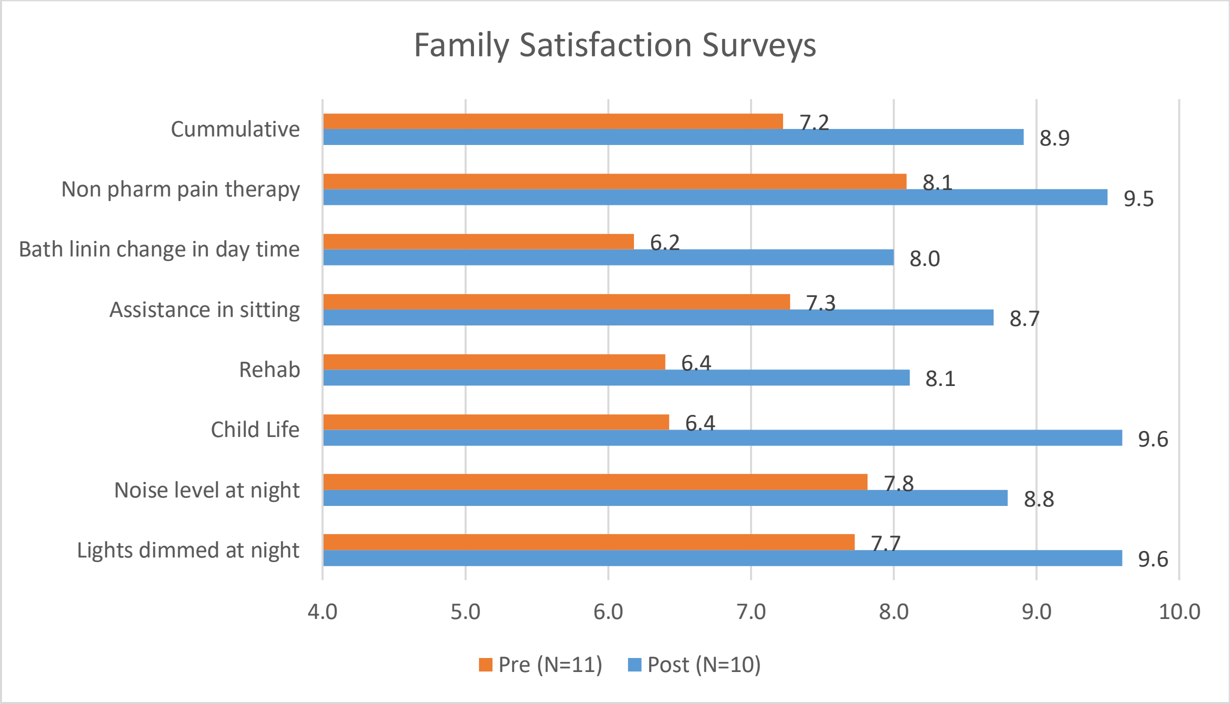

Median cumulative score pre 7.7 (IQR 5.3, 8.5) and post 8.8(IQR 8.2, 9.6), p=0.01 (Wilcoxon test)
